# Associations Between Executive Functions and Physical Fitness in Preschool Children

**DOI:** 10.3389/fpsyg.2021.674746

**Published:** 2021-08-02

**Authors:** Aleksander Veraksa, Alla Tvardovskaya, Margarita Gavrilova, Vera Yakupova, Martin Musálek

**Affiliations:** ^1^Psychological Institute of Russian Academy of Education, Moscow, Russia; ^2^Department of Psychology, Lomonosov Moscow State University, Moscow, Russia; ^3^Institute of Psychology and Education, Kazan Federal University, Kazan, Russia; ^4^Faculty of Physical Education and Sport, Charles University, Prague, Czechia

**Keywords:** cognitive flexibility, working memory, inhibitory control, physical fitness and sport, motor performance

## Abstract

Considering the current agreement on the significance of executive functions, there is growing interest in determining factors that contribute to the development of these skills, especially during the preschool period. Although multiple studies have been focusing on links between physical activity, physical fitness and executive functions, this topic was more investigated in schoolchildren and adults than in preschoolers. The aim of the current study was to identify different levels of physical fitness among pre-schoolers, followed by an analysis of differences in their executive functions. Participants were 261 5–6-years old children. Inhibitory control and working memory were positively linked with physical fitness. Cognitive flexibility was not associated with physical fitness. The research findings are considered from neuropsychological grounds, Jean Piaget’s theory of cognitive development, and the cultural-historical approach.

## Introduction

The transformations of modern lifestyle add special pertinence to the investigation of the role of children’s physical development. This involves the phenomena such as digitalization of the education, the loss of priority of traditional games, and the shifting structure of children’s communities (Linebarger et al., 2014; Lillard et al., 2015). Hypodynamia and the metabolic and cardiovascular problems linked to above-mentioned phenomena are not the only issues this study addresses, it also considers the influence on children’s mental development which has been shown in recent studies (Bames et al., 2012). The domain that is most strongly associated with physical development is the formation of executive functions (Diamond, 2000). Therefore, we will look closely at the links between them.

### Executive Functions

The exact definition of executive functions has been subject to discussion for a long time. In terms of our study, we rely on the following definition: “cognitive processes that are required for the conscious, top-down control of action, thought, and emotions, and that are associated with neural systems involving the prefrontal cortex” (Lerner et al., 2015, p. 271). The main executive functions parameters are three cognitive competences: (i) inhibitory control (resisting habits, temptations, or distractions); (ii) working memory (retaining and using information); and (iii) cognitive flexibility (Miyake et al., 2000; Nelson et al., 2016). executive functions competences develop intensively throughout the preschool years and have a significant effect on child’s later performance (Best and Miller, 2010).

Executive functions crucially impact the formation of academic skills such as word reading, vocabulary, spoken and written language comprehension, mastering of initial mathematical concepts, development of speech skills, as well as the outcomes in high school (Zelazo et al., 2003; Blair and Razza, 2007; Utendale et al., 2011; Blankson et al., 2012; Cheie et al., 2015; Torres et al., 2015; Gagne, 2017). Studies indicate the presence of individual variations in executive functions development by the time children start school (Liebermann et al., 2007; Garon et al., 2008; Lan et al., 2011). Children with considerably lower executive functions indicators are disadvantaged even before their first school year. This starting difference between the advantaged and the less advantaged only grows over time (Prencipe et al., 2011; Skogli et al., 2017; Lensing and Elsner, 2018). Considering the current agreement on the significance of executive functions in early years, there is growing interest in determining factors that contribute to the development of these skills, especially during the preschool period.

### Physical Activity and Physical Fitness

Physical fitness includes about eleven components which can be divided into two groups. The first group comprises health orientated fitness parameters such as muscle strength, power, muscle endurance, cardiorespiratory fitness, and flexibility. The second one spans skill-related physical fitness such as agility, coordination and speed (Corbin and Le Masurier, 2014). In order to investigate both areas of physical fitness in preschoolers, a number of complex tests was designed, generally including the same set of “core” tests: (1) standing broad jump which allows to assess the explosiveness of the lower limbs; (2) sit-ups to assess the endurance of the trunk muscles; (3) agility shuttle running to assess speed coordination; (4) flexibility tests, which usually include different variations of sit and reach exercises and are used to assess the flexibility of the lower back and hamstrings; and (5) a multistage fitness test, i.e., progressive 20 m shuttle running test designed to assess cardiorespiratory fitness (Council of Europe, 1988; Měkota and Kovář, 1995; Morrow et al., 2009; Meredith and Welk, 2010; Ruiz et al., 2011; Ortega et al., 2015). Typically, the physical fitness level is calculated based on a number of motor tests which evaluate physical readiness. The readiness is significantly associated with health benefits or risks, as well as to physical activity (US Department of Health and Human Services, 2008; Bermejo-Cantarero et al., 2017).

Physical activity was defined as follows in the study of Welk et al. (2000, p. 65): “Physical activity refers to any bodily movement produced by skeletal muscles that results in energy expenditure” (Caspersen et al., 1985). This formulation of physical activity bears resemblance with the physical fitness definition. Nonetheless, it needs to be noted that physical activity is a biological process which can considerably activate and stimulate children’s central nervous system to gain knowledge (Rowland, 1998) and possibly improve the development of cognitive functions (Pellegrini and Smith, 1998; Biddle and Asare, 2011; Carson et al., 2016). Thus, physical activity in children must not be evaluated using only physical fitness parameters, as was indicated by several studies (Welk et al., 2000; DeBate et al., 2009). To conclude, although both physical fitness and physical activity are important markers of both physical and mental health in children, the link between the two is not necessarily direct.

### Executive Functions and Physical Fitness

Physical activity and physical fitness are crucial to human development (Silva and Annamalai, 2008; Dumith et al., 2011; Rarick, 2012). These concepts are not only considered important parameters of physical health (Robinson et al., 2015), but are also strongly associated with cognitive development. A study of factors associated with executive functions competences and its correlates indicates that physical activity and physical fitness can be seen as a specific domain (Best, 2010). Both physical activity and physical fitness generate natural motivation, create lasting conditions of “challenge,” and minimize the impact of factors inhibiting executive functions: stress, boredom, lack of sleep, and oxygen (Diamond and Ling, 2016).

From the perspective of neurological and microstructural changes it was found that during physical activity (PA) a higher production of neurotropic factors like the brain derived neurotropic factor (BDNF) or the neurotropic growth factor (NGF) responsible for increasing of neuroplasticity in brain regardless of age and sex are present (Barde, 1989; Vaynman and Gomez-Pinilla, 2006; Shim et al., 2008) and positive changes in the white matter microstructure (Arvidsson et al., 2018; Chaddock-Heyman et al., 2018) occur. These neural growth changes along with altered synaptic transmission and induction of brain vascularization during physical activities (e.g., fitness, motor competence actions) influencing particularly prefrontal cortices responsible for thinking, decision making, or behavior were summarized under the executive function hypothesis (Kopp, 2012; Diamond, 2013).

In the last four decades research has proved that physically active breaks during a school day (Grieco et al., 2009), or different movement/physical interventions lasting from 10 to 40 min (Ellemberg and St-Louis-Deschênes, 2010; Kamijo et al., 2011) have a moderate to strong positive effect on increasing of on-task behaviors, attention, or working memory in pre-adolescents and adolescents. Although multiple studies have been focusing on links between physical activity, physical fitness and EF, this topic was more investigated in schoolchildren and adults than in preschoolers (Dwyer et al., 2001; Colcombe and Kramer, 2003; Sibley and Etnier, 2003; Planinsec and Pisot, 2006; Davis et al., 2007; Roebers and Kauer, 2009; Fedewa and Ahn, 2011; Verburgh et al., 2014). Number of studies documented positive links between physical activity and cognitive performance in preschool age children (Sibley and Etnier, 2003; de Greeff et al., 2018). Further, previous research pointed on significant relation between physical activity and physical fitness (Latorre-Román et al., 2016). Therefore, the degree of physical fitness in preschoolers might be candidate significantly related to the degree of cognitive or executive functions (Visier-Alfonso et al., 2020). Even though, recent studies support this assumption (Maurer and Roebers, 2019; Visier-Alfonso et al., 2020) the role of physical fitness and its certain components (a) muscular and (b) motor component in development of executive functions skills is still open issue.

García-Hermoso et al. (2020) found a significant relationship between the effect of intervention and attention and concentration when changing in cardiorespiratory fitness. However, the authors note a significant limitation of the study, which is the lack of other specific cognitive abilities that composed executive functions. Mulvey et al. (2018) revealed that gross motor skills program led to significant improving of executive functions in preschool children. Oberer et al. (2018) found interrelations between visual and motor coordination and reading and counting skills. Further, in this study it was suggested that the positive influence of physical fitness on academic achievement is substantial but indirect via executive functions. Chang et al. (2012) concluded that motor skills are a predictor of cognitive flexibility and working memory, and they in turn predict the development of reading skills with a high degree of reliability in preschool age children 5–6 years old. On the other hand, recent study of Houwen et al. (2017) did not support this finding in children 3–4 years. The authors investigated the relationship between three variables: motor skills, executive function and passive vocabulary in children aged 3–4 years.

Review of the research indicates that it mostly considered the association between physical activity and executive functions in preschool-age children, while the connection between physical fitness and executive functions is still insufficiently understood. This can be due to the fact that children’s physical activity can be investigated using survey methods that are not challenging in its application, while the study of physical fitness necessitates the in-person examination of children, a suitable sports facility, and setting up all the equipment required for the tests. In our view, investigating links between physical fitness and executive functions can be more informative for a number of reasons. First, data that is collected on physical fitness is more objective, since it is acquired in an in-person examination of children, rather than through a survey of parents. This is confirmed by the inadequacy of survey-based methods in studying child development (McDonald, 2008). Second, even regular sports activities over a long period of time don’t necessarily lead to a high level of physical fitness, because children may skip classes, the activities may be inadequate for their age and capabilities, or aimed only at specific physical fitness indicators.

Given that the positive influence of physical fitness on the executive and cognitive functions seems to benefit brain development, our study was designed to verify whether the physical fitness in pre-schoolers were significantly linked to their performance in specific executive functions. Following hypotheses were formulated to explore these relationships: (1) the results from the five physical fitness tests will constitute a two-factor structure composed of the physical fitness and a motor skill construct; and (2) high level of physical fitness positively relates to main executive functioning skills among children. Thus, the main purpose of the study is to identify different levels of physical fitness among children aged 5–6 years, followed by an analysis of differences in their EF. This research question is whether there are any differences in children’s executive functions performance depending on their physical fitness measures separately or on the general physical fitness status.

### Method

#### Participants

Participants were 261 typically developing 5–6-year-old (*M* = 5.77, SD = ± 0.32) children (boys *n* = 130, girls *n* = 131) from primarily medium-income families. Boys and girls in our sample non-significantly differed in their mean age: boys $\bar{x}=5.79\pm 0.33$; girls $\bar{x}=5.76\pm 0.31$; *t* = 0.76, *p* = 0.45. Children were attending four various pre-kindergarten classrooms located in Moscow. Assessment of executive functions was carried out individually with each child. This study and consent procedures were approved by the Ethics Committee of Faculty of Psychology at Lomonosov Moscow State (approval no. 2020/72). All parents provided written informed consent for their child’s participation in the study.

#### Executive Functions Measures

*The Dimensional Change Card Sort (DCCS*, Zelazo, 2006) is an executive functions task aimed at measuring cognitive flexibility. In the DCCS task, a child is asked to sort cards in three rounds, according to different rules. The first sorting is based on the picture’s color (pre-switch trial), the second on shape (switch trial), and the third on conflicting rules: on color or shape of card depending on is their presence of frame on the card or not (post-switch trial). In the analysis we used the final score of the methodology with a range of scores from 0 to 24.

The subtest *Inhibition* (Korkman et al., 2014) is an executive functions task that assesses the child’s ability to inhibit automatic cognitive responses. It includes two series of shapes (circles/squares, and arrows). Firstly, the child is asked to name the shape or direction (Naming trial). In the second part of the task, a child is asked to name the shape or direction conversely: to name circles when squares are presented and squares when circles are presented (Inhibition trial).

The subtest *Sentences Repetition* (Korkman et al., 2014) aimed to assess verbal working memory. This technique uses 17 sentences, gradually increasing in their complexity (sentences become longer and syntactically more complex). For example, while the first sentence consists of 2 words and has a simple structure – “Good night,” the twelfth sentence consists of 14 words and has a complex structure – “The woman, who stands next to a man in a green jacket, is my aunt.” Omitting a word, replacing it or adding another word was considered as an error. Changes in word order, as well as word relocation, were also considered as an error. An accurately reproduced sentence received 2 points, a sentence containing 1 or 2 errors received 1 point, a sentence with 3 errors or more received 0 points. If a child received 0 points for four consecutive sentences, then the test was terminated.

The subtest *Memory for Designs* (Korkman et al., 2014) aimed to assess visual working memory. Two parameters of visual memory were measured – memorization of “pictures” (selection of pictures, as in a presented sample, from an array of similar pictures) and memorization of a spatial arrangement of the pictures (recall the cards’ position in a sample). For each task, 2 points were awarded for each correctly chosen card (called “Content score”) and 1 for each correctly indicated place (called “Spatial score”). Two bonus points were given on each trial if a child correctly selected the card and placed it on its right place (called “Bonus score”). As a result, four estimates were obtained for visual working memory: a content score, a spatial score, a bonus score and a total score (sum of all points in all tasks), as described in the NEPSY-II battery.

All methods have been adapted and validated in the Russian sample and have shown high psychometric qualities (Almazova et al., 2020). Trained researchers measured the variables and outcomes of the study under standardized conditions. All data were collected at the same time in the morning, between 8:00 am and 11:00 am. All values were converted to Z-scores.

#### Physical Fitness Measures

According to one of the most widely used models – the Stodden model – the physical fitness is composed from the muscular component (power or explosive strength, isometric strength, muscular endurance) and the motor skill component (agility, balance, coordination, speed of movement) (Bouchard et al., 1994; Malina et al., 2004). This study has attempted to cover both components. Thus, four physical fitness tests were used to asses physical fitness. All of them have been determined based on the principle of having relevant physical activity in children’s everyday lives.

##### Broad jump

Standing behind the starting line with legs slightly apart (feet shoulder width apart), the participant will flex the knees and extend the body forward as arms extend behind the body. Extending the arms forward and then upward, the participant will take off and jump as far as he can. The length of the jump in centimetres is evaluated, each participant has 3 attempts. The distance is measured from the starting line to the rear edge of the last footprint. The motor task is explained and demonstrated.

##### Sit and reach test

The child is asked to sit down against the wall, straighten the lower limbs and lean against the wall with the whole back. The child is then asked to straighten the back. The modified bench which is 25 centimetres high (about 5 centimetres lower against original sit and reach bench) is moved to the legs (feet), so that the entire surface of the child’s feet rests on one side of the bench. Subsequently, the child is asked to stretch the arms forward. The metric scale is moved to the tip of the middle finger. Then the child performs a forward bend. While bending, the child must not flex the knees of the lower limbs. The examiner checks this by holding his hand on the child’s knees throughout the whole trial. The maximum distance the child reaches without breaking knees for 2 s is recorded. The child performs the entire test twice, in rapid succession.

##### Shuttle run 4 × 5 meters

For the test, a distance of 5 m must be marked. The first base (a colored cone) is located 20 cm before the starting line, the second cone is located at a distance of 5.2 m. The participant starts from a semi-crouched start and starts running following the signal, touches the top of the cone and returns. Here he touches the second cone, again runs to the cone he touched first colored cone and touch it for second time. Then the participant runs back and must touch the finish (starting) colored cone. Each child has a trial run, after that each participant has two attempts. The time the participant needed to run the whole track is recorded. The time is recorded on the stopwatch and the time is stopped when the participant touches the finish (starting) colored cone.

##### Throwing a tennis ball

Children are given a tennis ball (always start with the right hand). The child has to throw by overhead technique the ball as far as possible. The examiner demonstrates the execution of the throw. Each child throws the ball three times with the right hand and three times with the left hand. The throws follow immediately one after another. The examiner checks that the child does not step on the line representing the starting line for the throw. He also observes whether the child stands in a cross position of the lower and upper limbs, but does not correct the position if the child stands in a different position.

#### Data Analysis

Since we have results from both sexes from tests which are sex dependent in the age range overcoming one age category (5.01 to- 6.50 years) we used raw scores from test only to show dependency between sex and Executive function degree and Physical fitness level applying appropriate type of parametric or non-parametric *T*-test. For other analyses we have converted all values regarding age and sex to Z-scores. Firstly, we tested whether the used motor tests were significantly related to the physical fitness constructs. For the purpose of this investigation, we conducted a confirmatory factor analysis. Since the Mardia test, the Henze-Zirkler’s test and the Royston’s test rejected multivariate normality of the used motor tests we used maximum likelihood with a robust standard errors (MLR) estimation parameter for verification of the proposed two factor structure. For assessing the quality of the suggested model we used the recommended fit indices along with the suggested cut offs: (1) model discrepancy: Chi-square (S-BX2), model significance *p* > 0.05; (2) approximating error: Root Mean Square Error of Approximation (RMSEA) <0.06, Standardized Root Mean Square Residual (SRMR) ≤0.08; and (3) incremental fit indices: Comparative Fit Index (CFI) >0.95, Tucker-Lewis Index (TLI) >0.95 McDonald, 1999; Maydeu-Olivares, 2006; McDonald and Marsh, 1990). To investigate the main effect between physical fitness and each component of the cognitive profile we used one-way non-parametric ANOVA. The Duncan *post hoc Z*-test for concrete differences between groups was used with cut off >2.39. All statistical procedures were carried out in Mplus6 and NCSS2007 (Hintze, 2007) software.

### Results

#### Factor Structure of Physical Fitness Construct

**FIGURE 1:**
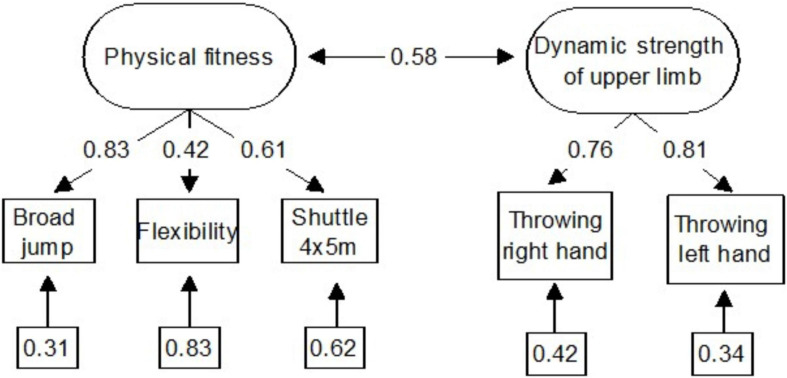
Path diagram two-factors structure of physical fitness indicators.

In the first step, we tested structural hypothesis that the results from the five used physical fitness tests will constitute a two-factor structure composed of the physical fitness and a motor skill construct. Results of two confirmatory factor analyses (CFA) showed that all four used tests fit well the suggested two factor model with moderately correlated *r* = 0.58 factors: (a) physical fitness (broad jump, sit and reach, 4×5 m shuttle running) and (b) dynamic strength of upper limb and trunk (throwing), instead of a unidimensional structure (S-B*_*X*_^2^* = 0.59, DF = 4, RMSEA = 0.00, RMSEA 90% C.I. = 0.000 – 0.000, CFI = 1.00) (see Figure 1 and Table 1).

**TABLE 1:** Unidimensional and two factor model of physical fitness construct.

**Model**	***N***	**S-B*_*X*_^2^***	***p***	**DF**	**RMSEA**	**RMSEA 90% C.I.**	**SRMR**	**CFI**	**TLI**
									
One factor model	261	68	0.000	5	0.22	0.175 – 0.268	0.074	0.77	0.54
Two factor model	261	0.59	0.96	4	0.00	0.000 – 0.000	0.009	1.00	1.03

Previous research found that executive functions development as well as physical fitness development are sex dependent. Therefore, in the next step we compared the results of boys and girls achieved in executive function tests and physical fitness tests.

All executive function tests displayed non-normal data distribution. Therefore, median statistics Mann Whitney *U* test was used for comparison. Despite, for better readability we decided to present mean and standard deviation values in Table 1. From the results of the executive function tests (see Table 2) it is evident that girls significantly outperformed boys with the largest differences in the area of working memory. Only in Inhibition trial (Corrected error) no significant difference between boys and girls was found. Since the physical fitness tests passed normal distribution requirements, the parametric two sample *T*-test was applied. Boys performed significantly better in agility and dynamic strength of trunk and upper limb fitness tests, while girls significantly outperformed boys in flexibility.

**TABLE 2:** Sex differences in executive function and physical fitness performance.

**Items**	**Boys Mean ± SD**	**Girls Mean ± SD**	***z*-value**	***p***
**Executive functions**				
Cognitive flexibility (DCCS score)	18.12.9	19.32.7	3.45	< 0.001***
Naming trial (Uncorrected Errors)	0.81.4	0.61.4	2.2	0.02*
Naming trial (Corrected Errors)	1.11.2	0.91	2.18	0.02*
Naming trial (Time)	46.313.4	42.510	2.1	0.02*
Inhibition trial (Uncorrected Errors)	4.36.6	2.95.6	2.6	0.002**
Inhibition trial (Corrected Errors)	2.31.9	1.91.6	1.4	0.09
Inhibition trial (Time)	60.815.7	57.112.8	2.1	0.02*
Visual working memory (Content Score)	37.26.6	40.85.6	4.3	< 0.001***
Visual working memory (Spatial Score)	18.44.6	20.34.2	3.2	< 0.001***
Visual working memory (Bonus Score)	18.912.8	22.914.3	2.2	0.02*
Visual working memory (Total Score)	74.522	83.821.4	3.4	< 0.001***
Verbal working memory (Total Score)	184.9	20.14.9	4.0	< 0.001***
**Physical fitness**				
Running 4×5 m s	9.51	9.70.7	2.52	0.012*
Broad jump cm	100.818.8	97.818	1.02	0.15
Flexibility cm	13.45.7	17.46.3	5.40	< 0.001***
Throwing right hand cm	612165	520142	4.80	< 0.001***
Throwing left hand cm	478140	403120	4.70	< 0.001***

*p < 0.05 (2-tailed), **p < 0.01 (2-tailed), ***p < 0.001 (2-tailed).

#### Differences in Executive Functions Depending on the Level of Physical Fitness

Further, the Z-scores of each participant on the motor test were weighted by its factor loading received from the two-factor structural model. We designated it as the composite physical fitness score. According to this composite score, we separated the children into three groups:

(1) Below-average physical fitness: ≤25th centile *n* = 78 (boys *n* = 38, girls *n* = 40) age $\bar{x}=5.75\pm 0.32$ years
(2) Average physical fitness: 26 – 74th centile *n* = 113 (boys *n* = 55, girls *n* = 58) age $\bar{x}=5.75\pm 0.31$ years
(3) Above-average physical fitness: ≥75th centile *n* = 70 (boys *n* = 37, girls *n* = 33) age$\bar{x}=5.85\pm 0.33$ years

In further analysis, the differences in the defined cognitive areas considering the centile group based on composite physical fitness score in all children regardless of their sex were compared. Since the results in Table 2 showed significant differences in executive functions performance between boys and girls, we also converted all raw scores obtained from executive function tests into Z-scores respecting sex differences. As it was found that the data from executive function tests were not normally distributed, we used non-parametric Kruskal Wallis Analysis of Variance (see Table 3).

**TABLE 3:** Differences in results of executive functioning test between three defined groups of children based on the composite physical fitness score.

	**Under-average Median Z-score**	**Average Median Z-score**	**Above-average Median Z-score**	**Chi-square**	***p***
Cognitive flexibility (DCCS score)	–0.08	–0.02	0.03	4.29	0.12
Naming trial (Uncorrected Errors)	−0.41^(a)^	–0.21	0.41	6.78	0.03*
Naming trial (Corrected Errors)	0.12	0.12	0.81	5.7	0.06
Naming trial (Time)	−0.08^(b)^	0.25^(b)^	0.48^(b)^	19.52	< 0.001***
Inhibition trial (Uncorrected Errors)	–0.35	–0.50	–0.35	2.45	0.29
Inhibition trial (Corrected Errors)	–0.15	0.05	–0.15	1.37	0.50
Inhibition trial (Time)	–0.12	0.32^(c)^	0.41^(c)^	12.17	0.002**
Visual working memory (Content Score)	–0.19	–0.15	0.12	1.78	0.41
Visual working memory (Spatial Score)	–0.24	0.24^(c)^	0.39^(c)^	12.7	0.002**
Visual working memory (Bonus Score)	–0.51	0.21^(c)^	0.07^(c)^	11.20	0.004**
Visual working memory (Total Score)	–0.15	0.21	0.34	8.36	0.02*
Verbal working memory (Total Score)	–0.23	–0.02	0.18	6.47	0.03*

^(a)^Significant difference between above-average and under-average motor competencies groups. ^(b)^Significant differences between all three groups of children. ^(c)^Significant differences between above-average and average and under-average motor competencies groups; no significant difference between average and under-average groups.*p < 0.05 (2-tailed), **p < 0.01 (2-tailed), ***p < 0.001 (2-tailed).

Analyses revealed significant differences in the degree of physical fitness in inhibition (Naming Uncorrected Errors; Naming Time and Inhibition Time) and the working memory variables (both visual and verbal). Furthermore, a *post hoc* Dunn’s test analysis revealed that children with above-average physical fitness achieved significantly better scores in visual and working memory. These children also made significantly fewer mistakes in the test involving inhibition control (Naming Uncorrected Errors) than peers in the below-average motor competency group.

The tests Naming trial (Time) and visual working memory (Bonus and Total Scores) were even more sensitive to the degree of the children’s motor competencies. In these tests, children with above-average and average motor competencies scored significantly better than children with below-average motor competencies. The Naming time variable in the inhibition task produced the greatest differences in executive functioning performance, considering the degree of motor competencies. In this test, a *post hoc* Dunn’s test showed significant differences between all three groups of children. The children with an above-average degree of motor competencies had the best scores.

#### Differences in Physical Fitness Depending on the EF

Further, we investigated whether performance in each physical fitness test is differently related to EF. Therefore, we divided, according to the same rules converting into Z-scores, children into three groups: (1) Under average performance ≤25th centile, (2) Average performance 26th – 74th centile, and (3) Above average performance ≥75th centile, for each physical fitness test. Table 4 shows the distribution of frequencies in these three categories considering sex.

**TABLE 4:** Frequency of under-average, average and above-average physical fitness performance in each test.

	**Under-average performance**		**Average performance**		**Above-average performance**	
	**Boys**	**Girls**	**Boys**	**Girls**	**Boys**	**Girls**
Broad jump	*n* = 37	*n* = 48	*n* = 51	*n* = 39	*n* = 41	*n* = 45
Flexibility	*n* = 41	*n* = 47	*n* = 49	*n* = 41	*n* = 39	*n* = 44
Shuttle run	*n* = 26	*n* = 41	*n* = 64	*n* = 45	*n* = 39	*n* = 46
Throw right	*n* = 44	*n* = 40	*n* = 40	*n* = 51	*n* = 46	*n* = 40
Throw left	*n* = 39	*n* = 41	*n* = 51	*n* = 54	*n* = 39	*n* = 37

The main point was to find whether the association between executive functions tests and physical performance could be dependent on the movement character of the motor test. Therefore, Table 5 shows in which of physical fitness tests the performance level is significantly related to performance in executive functions tests previously found as significantly associated with PF.

**TABLE 5:** Differences in physical fitness performance depending on Naming shape time.

	**Under-average median**	**Average median**	**Above-average median**	**Chi-square**	***p*-value**
**Naming shape time**					
Broad jump	–0.12	0.29^(c)^	0.43^(c)^	11.13	0.004***
Flexibility	0.08	0.39^(c)^	0.36^(c)^	12.33	0.002**
Shuttle run	0.13	0.28	0.43^(a)^	7.26	0.03*
Throw right	0.18	0.15	0.41^(a)^	8.18	0.02*
Throw left	0.08	0.22^(c)^	0.46^(c)^	10.39	0.006**
**SR**					
Broad jump	−0.30^(d)^	0.03	0.18	8.83	0.01**
Flexibility	−0.20^(d)^	–0.07	0.30	7.93	0.02*
Shuttle run	0.28^(d)^	0.13	0.43	7.26	0.03*
Throw right	0.18	0.15	0.41	8.18	0.02*
Throw left	0.08^(d)^	0.22	0.46	10.39	0.006**
**Spatial**					
Shuttle run	–0.03	0.14	0.43	7.08	0.03*
Throw right	−0.27 ± 1	0.17 ± 1.02	0.51	7.28	0.03*
Throw left	0.01	0.26	0.50	8.24	0.02*
**Bonus**					
Shuttle run	–0.45	–0.12	0.01	6.79	0.03*
Throw right	–0.3	–0.09	–0.02	8	0.02*
Throw left	–0.45	–0.18	0.22	11.39	0.004**
**Total score**					
Shuttle run	–0.38	–0.19	–0.09	6.97	0.03*
Throw right	–0.39	0.07	0.02	7.2	0.03*
Throw left	–0.46	0.02	0.27	10.15	0.006**

^(a)^Significant difference between above-average and under-average motor competencies groups. ^(c)^Significant differences between above-average and average and under-average motor competencies groups; no significant difference between average and under-average groups. ^(d)^Significant difference between above-average and under-average motor competencies groups.*p < 0.05 (2-tailed), **p < 0.01 (2-tailed), ***p < 0.001 (2-tailed).

A detailed analysis presented in Table 5 showed the strongest association between performances in physical fitness tests and Naming shapes time. Performance in this test was positively associated with performance in all five physical fitness tests. A *post hoc* analysis revealed that children with above-average performances in these motor competency tests achieved significantly better results in Naming shapes time compared to children with under-average performance. Moreover, children with average performances in flexibility and throwing with right as well as left hand achieved significantly better results in Naming shapes time compared to peers with under-average performances in motor competency tests. Children with under-average performance in all physical fitness tests scored worse in verbal working memory test (Sentences Repetition) compared to counterparts with above-average performance. Moreover, in Throwing with left hand also children with average performance scored significantly better in SR compared to counterparts whose performance in Throwing with left hand was under-average. Visual working memory (Memory for Design Total Score) showed a significant relation with three motor competency tests. Children who achieved above-average performances in tests which demanded higher coordination, such as shuttle run and throwing with either right or left hand, scored significantly better compared to counterparts with under-average results (see Table 5).

#### The Role of Physical Fitness in Executive Functions Performance for Girls and Boys

The level of physical fitness and the outcomes in executive functions tests are not univariate in gender. As it was found that the data from executive function tests were not normally distributed, and a non-parametric Two-way Analysis of Variance (ANOVA) is not available, we used a non-parametric One-way ANOVA for each sex separately. The level of physical fitness was shown to play a significant role both for boys and girls in two executive functions variables: Naming trial (Time) and Visual working memory (Spatial Score). In Naming trial (Time) girls and boys with above-average physical fitness scored significantly higher than children with average and below-average physical fitness (boys Chi-square = 8.50 *p* = 0.014); (girls Chi-square = 18.32 *p* < 0.001) which was more pronounced in girls. In visual working memory, boys with above-average physical fitness scored significantly higher than boys with average and below-average physical fitness (boys Chi-square = 6.01 *p* = 0.049). In girls, *post hoc* Dunn’s test indicated that girls with below-average physical fitness scored significantly lower than girls with above and average physical fitness (girls Chi-square = 9.74 *p* = 0.008).

Significant links between the level of physical fitness and Inhibition (Corrected Errors), Inhibition (Time), Visual working memory (Bonus Score), Visual working memory (Total Score) were only shown in girls. In Z-Inhibition trial (Corrected Errors) score, girls with average physical fitness achieved significantly lower results than girls with above-average or below-average physical fitness (girls Chi-square = 8.48 *p* = 0.014). In Z-Inhibition trial (Time), girls with average and above-average physical fitness scored significantly higher than girls with physical fitness below-average (girls Chi-square = 6.65 *p* = 0.036). Unlike in boys, in the girls’ sample, the physical fitness level played a significant role in Visual working memory (Bonus Score). Girls with above-average physical fitness performed significantly better than girls with average and below-average physical fitness (girls Chi-square = 12.17 *p* = 0.002). Moreover, girls with above average and average physical fitness scored significantly higher on Visual working memory (Total Score) than girls with below-average physical fitness (girls Chi-square = 11.65 *p* = 0.003).

## Discussion

Theories and studies on physical fitness significantly contributed to our understanding of the strategies to improve executive functioning in children. One of the important paths for further investigation of executive functions in preschoolers should lead to elaboration of specific evidence-based strategies to improve their executive functioning skills. Studies confirm the potential impact of physical activity and physical fitness on the development of the inhibitory control and visual and verbal working memory which might be significantly linked to changes in the production of neurotrophic factors such as NGF and BDNF, which are responsible for the development of neural networks.

One of the research goals was to verify hypothesis concerning a two-factor structure of physical fitness in preschoolers. Confirmatory factor analysis confirmed that the data collected is compliant with the two-factor Stodden model, including physical fitness and motor competences. This outcome suggests that children’s physical fitness is not an elementary one-factor construct, but a more complex two-factor structure. This points to the need of using a number of tests both for physical fitness and a motor skill construct in terms of the physical fitness evaluation.

The next step was to test the hypothesis that high level of physical fitness positively relates to main executive functioning skills among children. Comparison of the executive function’s tests scores throughout all three groups with high, medium, and low overall physical fitness levels has shown a number of differences. The analysis has indicated that children with high level of total physical fitness generally score better than their peers with low or average total physical fitness level in relation to executive functioning. Hence, the most significant differences were found in the inhibitory control and in children’s ability of memorizing spatial location of new objects in the visual-spatial memory test. In respective tasks, the participants with above-average or average physical fitness performed significantly better than their peers with below-average motor skills. Also, children with above-average physical fitness scored significantly higher than their peers with below-average physical fitness. Therefore, it is likely that physical fitness positively influences children’s ability to deal with executive functioning tasks. No differences have been found in the development of cognitive flexibility depending on physical fitness. Previously conducted research has indicated that the relationship between executive functions and physical fitness is possibly bi-directional.

The analysis of physical fitness’s impact on children’s executive functions has shown that physical fitness significantly influences the results of boys and girls in two executive functions tests: Naming trial (Time) and Visual working memory (Spatial Score). In the Naming trial (Time) girls and boys with above-average physical fitness scored significantly higher than children with average and below-average physical fitness. Nonetheless, in some aspects of development, outcomes varied depending on gender. In boys, high level of physical fitness was significantly positively linked with memorizing the spatial positions of visual elements. Girls with good physical fitness scored lower in memorizing objects’ spatial positions. Additionally, a significant association between physical fitness and the indicators of inhibitory control, and the overall score on the visual-spatial memory assessment test was only found in girls. Even though girls with high physical fitness showed poorer results at memorization the spatial positions of objects, they performed better at the tests in general, comparing to the girls with medium and low physical fitness levels.

The relation between physical activity and the development of executive functions is typically explained through one of the three main hypotheses. The first one relies on the J. Piaget theory which asserts the mutual dependency of cognitive and physical development: well-developed motor skills enable children to engage more actively in the interaction with their environment, which positively influences their cognitive development (Piaget and Inhelder, 1966). That’s why Oberer underlines those sports activities not only require high motor coordination but also directly make children implement their executive functions competences (planning, focusing on a goal, adapting to changing environment) (Oberer et al., 2018). The second hypothesis has neuropsychological grounds: the results suggest that the same areas of the cerebral cortex are engaged during executive functions tests and physical exercises (Diamond, 2000). Moreover, earlier research also has indicated that physical activity stimulates the production of neurotrophic factors such as BDNF and NGF which are instrumental in developing neural networks (Cho et al., 2012; Roh et al., 2017). Higher level of physical activity, which is likely to lead to better physical fitness, turned out to be linked to a higher NGF level and indirectly associated with the level of BDNF (Arvidsson et al., 2018). Some researchers suggest that this indirect and more complex function of BDNF in relation to physical activity is due to the BDNF found in blood samples and stored in platelets, which could also play key role in maintaining the energetic balance (Cho et al., 2012). Nevertheless, it was demonstrated that the impact of physical exercise on the production of BDNF is positively linked to the executive functioning level (de Assis and Almondes, 2017). This neurobiochemistry hypothesis was also confirmed by Ghafori et al. (2018). They discovered that after a motor exercise sequence, both the serum level of BDNF and executive functions in children with dysgraphia were significantly increased.

Interpreting these outcomes in terms of the cultural-historical approach, we introduce a third hypothesis to explain the relationship between physical fitness and the development of executive functions in preschool children. Physical development is the process of learning to control one’s own body. In the course of the physical development, children improve their motor coordination and handle complex exercises with more and more success. It can be assumed, that this progress is partly determined by physiological mechanisms (maturation of cortical areas, oxygen supply), but also by assimilating cultural means. Cultural means can be described as mental representations that children use to control their movements. High-class athletes and their coaches use a particular technique based on this principle – training through visualization. It consists in working through all the elements of the movement in one’s head. Using the imagination, athletes go through the exercise mentally, preparing to perform it. We suggest that thoroughly imagining the precise execution of an exercise can help children to better master the series of movements and to better control them while actually performing it. Mental representations involved in executing complex exercises (such as a shuttle run or a broad jump) can be separated into two big groups: visual representations of the required movements and visual representations of the environment. The current study supports this view. Preschoolers with high levels of physical fitness have better visual-spatial working memory. They tend to mentally go through complex tasks step by step and form an image of their actions before carrying them out. It is also possible that visualizing the surrounding situation and their following actions not only allows children to better coordinate their movements but also helps with the performance anxiety, which can also impact children’s outcomes. Therefore, we are inclined to believe that the physical fitness and the executive functions are linked bi-directionally. This has been supported by empirical data from our study and other research (Piché et al., 2012, 2015; Howard et al., 2018). This seems to be consistent with the cultural-historical perspective. Well-developed executive functioning skills enable children to score higher during the physical fitness evaluation, as they include greater ability to retain movement sequences and form mental representations. And vice versa, sport activities not only allow children to better control their bodies but also positively influence the ability to regulate cognitive processes. Moreover, doing sports requires the ability to follow a regime and rules, to concentrate, and distribute one’s resources. These are the competences that will help children to manage their resources, for example, in school. In some measure, this can account for the executive functions’ role as a moderator in a relation between physical fitness and children’s academic outcomes.

## Conclusion

This study was designed to identify different physical fitness levels among 5–6-year-old children and then analyze the differences in their executive functions. The study verified a two-factor model including physical fitness and motor competences in preschoolers and indicated that two of the three main components of executive functions – inhibitory control and both visual and verbal working memory – are positively related to children’s physical fitness. The greatest contribution to the differences in inhibitory control and both visual and verbal working memory among children by shuttle running and throwing, which have highest demanded for CNS in coordination of movement.

The study has the advantage of providing a comprehensive investigation of both executive functions and physical fitness rather than examining selected indicators, and of novelty in analyzing the contribution of different physical skills executive functioning in girls and boys. A limitation of the study are the rather small sample size and its cross-sectional design, which to some extent limits the reliability and depth of the conclusions. Further longitudinal analyses of the relationship between physical fitness and executive functions are planned for which data are already being collected as part of the project. However, despite these limitations, the study findings contribute to clarification of the relationship between the main executive functions and physical fitness skills in preschoolers, and verified a two-factor Stodden model structure which can be easily and repeatedly used to assess physical fitness and motor competences in kindergarten environment. The findings can be of great practical importance in the educational environment and, in particular, for the development of school curricula or the selection of the most effective physical education tasks aimed at promoting the development of preschool children’s executive functioning.

## Data Availability Statement

The raw data supporting the conclusions of this article will be made available by the authors, without undue reservation.

## Ethics Statement

The studies involving human participants were reviewed and approved by the Committee of Faculty of Psychology at Lomonosov Moscow State (approval no. 2020/72). Written informed consent to participate in this study was provided by the participants’ legal guardian/next of kin.

## Author Contributions

AV was responsible for research planning and organization of research activities (ethical approvals, contracting, and funding). He was actively involved in writing the manuscript. MG was responsible for organizing individual diagnostics of children, preparing data for analysis, and writing part of the text. VY was involved in the process of individual diagnosis of children and contributing to the writing of the text. MM supervised the research, participated in the planning and design of the study, worked with data, completely carried out statistical analysis, and participated in the writing of the text. All authors contributed to the article and approved the submitted version.

## Conflict of Interest

The authors declare that the research was conducted in the absence of any commercial or financial relationships that could be construed as a potential conflict of interest.

## Publisher’s Note

All claims expressed in this article are solely those of the authors and do not necessarily represent those of their affiliated organizations, or those of the publisher, the editors and the reviewers. Any product that may be evaluated in this article, or claim that may be made by its manufacturer, is not guaranteed or endorsed by the publisher.
